# Lymph Drainage in Pregnant Women

**DOI:** 10.1155/2013/364582

**Published:** 2013-10-22

**Authors:** Sylvia Cataldo Oportus, Lilian de Paiva Rodrigues, José Maria Pereira de Godoy, Maria de Fátima Guerreiro Godoy

**Affiliations:** ^1^Post Graduation Lymphovenous Rehabilitation Course, Medicine School of São Jose do Rio Preto (FAMERP), São Jose do Rio Preto, SP, Brazil; ^2^Obstetrics Outpatient Clinic of Santa Casa de São Paulo, São Paulo, SP, Brazil; ^3^Cardiology and Cardiovascular Surgery Department, Medicine School of São Jose do Rio Preto (FAMERP), São Jose do Rio Preto, SP, Brazil; ^4^Godoy Clinic Research Group and Medicine School of São Jose do Rio Preto (FAMERP), Avenida Constituição, 1306 São Jose do Rio Preto, SP, Brazil

## Abstract

*Aim*. The aim of this study was to evaluate the efficacy of lymph drainage to reduce edema of pregnant women. *Method.* Pregnant women (30 limbs) from the Obstetrics Outpatient Clinic of the Medical School of Santa Casa in São Paulo in the period December 2009 to May 2010 were enrolled in this quantitative, prospective study. The patients, in the 5th to 8th months of gestation, were submitted to one hour of manual lymph drainage of the legs. The volume of the legs was measured by water displacement volumetry before and after one hour of drainage using the Godoy & Godoy manual lymph drainage technique. The paired *t*-test was used for statistical analysis with an alpha error of 5% being considered significant. *Results*. Manual lymph drainage significantly reduced swelling of the legs of pregnant women during the day (*P* = 0.04). *Conclusion*. Manual lymph drainage helps to reduce limb size during the day of pregnant women.

## 1. Introduction

During pregnancy, many changes occur in the female organism with the adaptation for the fetus causing numerable complaints, for example, edema of the lower limbs. Peripheral edema is the most common and resilient manifestation in pregnant women. Its etiology includes the retention of sodium and water and changes in the circulation related to the effect of the gravid uterus on the inferior vena cava [1].

Moreover, during pregnancy, many hormonal changes take place including increased levels of progesterone, estrogen, HCG, and prolactin [2]. These higher levels of hormones induce changes in vascular permeability, promoting extravasation of plasma with consequent edema. Other transformations that may occur due to these hormonal changes are the formation of varicose veins, sensation of heaviness, paresthesia, and cramp. The prevalence in the general population of varicose veins is 56% for men and 60% for women with risk factors including age and number of pregnancies [3]. Treatment of varicose veins is usually divided into three types: surgery to remove the veins, medications, and nondrug therapy such as compression stockings. Medications or stockings are used to reduce the symptoms of swelling. One randomized study compared types of intervention used to relieve symptoms or treat lower extremity edema and varicose veins of 159 pregnant women. Sixty-nine women used hydroxyethylrutoside, 35 used elastic stockings and 55 were submitted to reflexology. Hydroxyethylrutoside seems to improve the symptoms of varicose veins, but it is not recommended as there are few studies evaluating its use during pregnancy. Reflexology has provided significant improvement in symptoms of edema; however, the number of patients reported in publications is very small. There are even fewer studies on the treatment of edema and varicose veins in pregnancy [4].

Nonpharmacological therapies such as lymph drainage may be used and seem to improve the swelling during pregnancy, but further studies assessing the efficacy are needed. One preliminary investigation used lymph drainage in the treatment of edema in pregnant women [5]. What comes is to reinforce the need for studies in this area. The aim of this study was to evaluate the efficacy of lymph drainage to reduce edema of women in the fifth to eighth months of gestation.

## 2. Method

Fifteen pregnant women (30 limbs) from the Obstetrics Outpatient Clinic of the Medical School of Santa Casa in Sao Paulo were enrolled in this quantitative, prospective study in the period from December 2009 to May 2010. The patients, with ages ranging from 23 to 38 years old (mean of 30.5 years), were in the 5th to 8th months of gestation. Participants were submitted to one hour of manual lymph drainage of the legs. The reduction in size was calculated by measuring the volume by water displacement volumetry before and after drainage.

The inclusion criteria were women in the 5th to 8th months of gestation who were suffering from edema due to the pregnancy and agreed to participate in the study. Women in high-risk pregnancies were excluded.

Randomization was by order of arrival at the clinic in this prospective, cross-sectional quantitative study. The order of the treatment and control days was by simple randomization.

Patients were evaluated for variations in edema and the effects of lymph drainage during the day.

Patients were evaluated on two days, one when lymph drainage was performed and the other without lymph drainage. Water displacement volumetry was performed at 7 to 8 o'clock a.m. and a second time between 2 and 3 o'clock p.m., thus, about 7 hours between the measurements. The Godoy & Godoy manual lymph drainage technique was utilized over one hour [6–9].

The paired *t*-test was used for statistical analysis with an alpha error of 5% being considered acceptable. The study was approved by the Research Ethics Committee of Santa Casa de Sao Paulo (number 324-09).

## 3. Results

Of the 15 participants, 51% were pregnant for the first time and 49% for the second.

The mean volume of the leg in the control evaluation was 2849.1 grams during the morning and 2889.1 grams in the afternoon, that is, a difference of +40.0 grams. On days that lymph drainage was performed, the mean initial volume was 2856.4 and in the afternoon it was 2812.0 grams, thus, giving a difference of −44.4 grams. The difference was statistically significant (*P* = 0.04, paired *t*-test) when compared to days without lymph drainage.

## 4. Discussion

This study demonstrated that manual lymph drainage reduces limb volume in pregnant women. Only one published study was found in the PubMed, ISI Web of Knowledge, and Scopus electronic databases evaluating lymph drainage as a means of reducing the volume of legs in pregnant woman [5].

Edema in pregnancy is common with the main therapeutic option being the use of elastic stockings; but this is not always tolerated by pregnant women. Medications are not always indicated with one of the few tested alternatives being micronized diosmin. Another alternative that has been evaluated in pregnancy is reflexology, although further studies are needed to test this as a therapeutic option [4].

The decision to evaluate lymph drainage throughout the day was made because of its indication in the treatment of edema. It stimulates physiological mechanisms of the lymphovenous drainage system favoring a reduction of edema. In this study the emphasis was to evaluate the pattern of swelling during the day. On comparing the mean volume on control days (without lymph drainage) with that when drainage was performed, there was a difference of about 80 grams. Thus, lymph drainage helps to maintain the limb size.

A study evaluating the change in volume of lower limbs of patients without clinical evidence of varicose veins (Clinical-Etiology-Anatomy-Pathophysiology-CEAP : C0 and C1) demonstrated that normal individuals have increases in the limb size during the day under normal conditions, and a compression stocking can prevent the limb from swelling [10].

The use of stockings associated with manual lymph drainage may have a synergistic effect on reducing swelling, but there is a need to evaluate this possibility.

The lymphatic system, works as a functional reservoir for the venous system, and edema occurs when the reserving capacity is exceeded. During pregnancy, the changes experienced by pregnant women favor the formation of edema. Therefore, the swelling is not due to lymphedema but due to system overload. Thus, the use of protective mechanisms is important during pregnancy.

## 5. Conclusion

Manual lymph drainage helps to reduce limb size during the day of pregnant women. Implications for nursing management: edema is the most common and resilient manifestation in pregnant women where there is a perceived need to treat or improve these symptoms in a clinic during this period; therefore, the outpatient nursing intervention with lymphatic drainage can effectively relieve this symptom afflicting these women.
